# Physical Origin of Dual-Emission of Au–Ag Bimetallic Nanoclusters

**DOI:** 10.3389/fchem.2021.756993

**Published:** 2021-09-27

**Authors:** Bo Peng, Liu-Xi Zheng, Pan-Yue Wang, Jia-Feng Zhou, Meng Ding, Hao-Di Sun, Bing-Qian Shan, Kun Zhang

**Affiliations:** ^1^ Shanghai Key Laboratory of Green Chemistry and Chemical Processes, College of Chemistry and Molecular Engineering, East China Normal University, Shanghai, China; ^2^ Laboratoire de Chimie, Ecole Normale Supérieure de Lyon, Institut de Chimie de Lyon, Université de Lyon, Lyon, France; ^3^ Shandong Provincial Key Laboratory of Chemical Energy Storage and Novel Cell Technology, School of Chemistry and Chemical Engineering, Liaocheng University, Liaocheng, China

**Keywords:** Bimetallic nanoclusters, pH ratiometric sensing, Structural water molecules, nanoscale interface, Dual-emission

## Abstract

On the origin of photoluminescence of noble metal NCs, there are always hot debates: metal-centered quantum-size confinement effect VS ligand-centered surface state mechanism. Herein, we provided solid evidence that structural water molecules (SWs) confined in the nanocavity formed by surface-protective-ligand packing on the metal NCs are the real luminescent emitters of Au-Ag bimetal NCs. The Ag cation mediated Au-Ag bimetal NCs exhibit the unique pH-dependent dual-emission characteristic with larger Stokes shift up to 200 nm, which could be used as potential ratiometric nanosensors for pH detection. Our results provide a completely new insight on the understanding of the origin of photoluminescence of metal NCs, which elucidates the abnormal PL emission phenomena, including solvent effect, pH-dependent behavior, surface ligand effect, multiple emitter centers, and large-Stoke’s shift*.*

## Introduction

Quantum-sized metal nanoclusters, which bridge the gap between organometallics and nanocrystals, exhibit dramatically unique electronic and optical properties, such as molecule-like energy gaps (Schaaff et al., 1997; Lee et al., 2004; Negishi et al., 2005; Walter et al., 2008; Zhu et al., 2008; Russier-Antoine et al., 2014; Bertorelle et al., 2017; Chakraborty and Pradeep, 2017), intense photoluminescence (Zheng et al., 2004; Zheng et al., 2007; Xie et al., 2009; Luo et al., 2012; Musnier et al., 2019) and catalytic properties (Tsunoyama et al., 2009; Zhu et al., 2010; Li et al., 2016a; Cai et al., 2019; Lv et al., 2019; Lv et al., 2020a; Yang et al., 2020a; Lv et al., 2020b; Cai et al., 2020; Li et al., 2021). Luminescent thiolated-protected Au and Ag NCs in particular have attracted tremendous interest due to their wide applications in bio-imaging, bio-medicine, sensing, and catalysis (Jin, 2010; Díez and Ras, 2011; Bonacic-Koutecky et al., 2012; Zheng et al., 2012; Luo et al., 2014; Sun and Jin, 2014; Jin et al., 2016; Lei et al., 2018; Yan et al., 2018; Ungor et al., 2021). Various strategies, such as heteroatom doping (Wang et al., 2014), aggregation-induced-emission (AIE) (Luo et al., 2012), and assembly induced emission enhancement (Wu et al., 2019a), have been developed to prepare highly luminescent Au and Ag NCs. Since the crystal structure of ligand-protected Au NCs, which are usually comprised of metallic core and peripheral gold(I)-thiolate staple motifs, have been revealed at atomic resolution (Jadzinsky et al., 2007), heteroatom substitution of specific native sites could give an in-depth way to understand the structure/composition-correlated properties and provide an efficient way to diversify and tailor the physicochemical properties of metal NCs (Hirai et al., 2020; Kang et al., 2020). Several strategies have been developed to the synthesis of bimetallic Ag-Au NCs, such as one-pot co-reduction method (Negishi et al., 2010; Kumara and Dass, 2011; Kumara and Dass, 2012) (spontaneous reduction of as-mixed Ag and Au precursors through balancing the redox potentials of metal pairs by thiol ligand (Dou et al., 2014a; Yu et al., 2016)), classical galvanic replacement reaction approach (Udayabhaskararao et al., 2012) (involves the spontaneous reduction of a noble-metal cationic by a less noble metal in solution driven by the difference in redox potentials), abnormal anti-galvanic replacement reaction approach (Choi et al., 2010; Wu, 2012) (inverse process for galvanic replacement reaction recently observed for the synthesis of thiolate-protected Ag-Au NCs), and addition reaction (Gan et al., 2018; Takano et al., 2018; Hirai et al., 2019) (a hydride-mediated controlled growth process). And intriguingly, as-formed heteroatom doped metal NCs generally exhibited dramatic enhancement of luminescent and catalytic performance (Dou et al., 2014b; Wang et al., 2014; Yao et al., 2015; Li et al., 2016b; Liu et al., 2017; Jana et al., 2019; Ye and An, 2019; Zhou et al., 2019), which are expected to have synergistic effects in their physicochemical properties compared with their mono-metallic analogues (Yuan et al., 2019). Nevertheless, the origin of PL or the nature of emitters of heteroatom-doped metal NCs remain unclear and even controversial, which limits the rational design of metal nanoclusters with improved and tailored optical and catalytic properties.

Luminescent metal NCs with dual-wavelength emission have been exploited as ratiometric nanoprobes for sensing and imaging (Xiao et al., 2018); rather than absolute intensity-dependent signal readout of single-emissive metal NCs, ratiometric measurements provide built-in self-calibration for signal correction, enabling more sensitive and reliable detection, which means the metal NCs possess two target-responsive reversible signal changes or that one of the signals is target-insensitive as reference (Liu et al., 2016; Huang et al., 2018; Wang et al., 2021a; Wang et al., 2021b). However, in most cases, well-resolved dual-wavelength emission of metal NCs was rarely observed simultaneously. Herein, we demonstrated that, using 1-dodecanethiol (DT) as a protected ligand, Au-Ag bimetallic NCs can be readily synthesized by a facile one-pot approach, exhibiting an interesting silver cation-mediated dual-emission behavior at 440 nm and 630 nm. Very interestingly, the dual-emissive Au-Ag bimetallic NCs display a unique reversible environment-pH-responsive emission behavior, which makes them ideal as potential pH ratiometric sensors: under acid conditions, the long-wavelength emission at 630 nm is dominated, while under basic conditions, the short-wavelength emission at 440 nm is prevailed. The combined characterizations of absorption, excitation, and emission spectrum evidenced that the dual-emissions come from the same luminous center, i.e., structural water molecules (SWs) dynamically confined on the metal NCs core, but with varied binding strength with surface Au(I)- and Ag(I)-thiolate motif, corresponding to the emission at 630 nm and 440 nm, respectively. The assignment of SWs as emitters is consistent with our previously reported results (Yang et al., 2020b; Hu et al., 2020), which also answers that the efficiency of dual-emissions is very sensitive to the delicate change of the surrounding microenvironment of metal NCs, including the dosing of Ag^+^, pH, and packing mode of surface ligands since the surface states formed by space overlapping of *p* orbitals of O atoms in the SWs {H_2_O∙OH^−^} as dynamic feature with *π* bonding nature.

## Method

### Synthesis

All reagents used were purchased from Sinopharm Chemical Reagent Co., Ltd. except glutathione in the reduced form (GSH), which was purchased from Aladdin (Shanghai, China). All chemicals were used as received without any further purification. Deionized water was used in all experiments.

*Synthesis of Ag*_*x*_ *Au@DT NCs with different ratio of Ag to Au.* The synthesis of luminescent Ag-Au NCs refers to modified literature reports (Ye and An, 2019). Typically, freshly prepared alcoholic solutions of DT (3.6 ml, 50 mM) and AgNO_3_ (x ml, 25.4 mM) were mixed well under vigorous stirring for 10 min, and then the solution was opacified due to the formation of Ag(I)-thiolate. Freshly prepared alcoholic solutions of HAuCl4 (0.2 ml, 25.4 mM) were then added into the mixture solution, the mixture was stirred for another 12 h, and then incubated overnight at room temperature. The feeding ratio of Ag to Au varied from 0 to 4, in which the volume of HAuCl_4_ precursor was constant but the volume of AgNO_3_ solution increased from 0 to 0.8 ml. The samples are denoted as Ag_x_Au@DT NCs, in which the x represented the feeding ratio of Ag to Au. The as-synthesized Ag_x_Au@DT NCs were used without purification.

Ultraviolet visible (UV-vis) spectroscopy was conducted with a UV2700 UV-vis spectrophotometer. Fluorescence was measured by using a FluorMax-4 fluorimeter (Horiba, Japan). HR-TEM images of NCs were collected with a JEOL JEM 2010 microscope operating at 200 kV. Fluorescence lifetime was measured with a homebuilt time-correlated single photon counting (TCSPC) system with a time resolution of sub-100 ps Phosphorescence lifetime was excited with a μF2 lamp and measured with FLS 980 spectrofluorimeter (Edinburgh Instruments). Thermogravimetric analysis (TGA) was conducted on a NETZSCH STA449F3 analyzer under air atmosphere (flow rate of 50 ml·min^−1^). Inductively coupled plasma (ICP) atomic emission spectroscopy was performed on a Thermo IRIS Intrepid II XSP atomic emission spectrometer. X-ray photoelectron spectra (XPS) were measured on an AXIS SUPRA instrument with X-Ray monochromatisation.

### pH Sensing

The stock solution of as-synthesized Ag_1_Au@DT NCs was acidic due to the reduction of Au (III) precursors and the formation of thiolate as the following two chemical equations:

$$\mathrm{HAuC}l_{4}\mathrm{+3 RS-H}\to \mathrm{4 HCl+A}u^{+}SR^{-}\mathrm{+RS-SR}$$ (1)

$$\mathrm{AgN}O_{3}\mathrm{+RS-H}\to \mathrm{HN}O_{3}\mathrm{+Ag+S}R^{-}\mathrm{+RS-SR}$$ (2)

The mole amount of H^+^ of the stock solution (4 ml) of as-synthesized Ag_1_Au@DT can be estimated to be about 0.025 mmol according to the equations. To evaluate the sensitivity of pH for as-synthesized Ag_1_Au@DT, 0.3 ml stock solution, which contained 1.875 nmol of H^+^, was diluted by adding 1.5 ml ethanol. Different volumes of 50 mM NaOH aqueous solution were used (0, 10, 15, 20, 25, 30, 35, 40, and 45 ul corresponding to 0, 0.5, 0.75, 1.0, 1.25, 1.5, 1.75, 2.0, and 2.25 nmol of OH^−^, respectively). The photoluminescence emission spectra of OH^−^ adjusted Ag_1_Au@DT NCs solution after standing overnight were record. The cyclic switching of the pH regulated PL intensity were performed by adding 50 ul 50 mM NaOH and 50 ul 50 mM HCl aqueous solution circularly. The solution of each cycle was ultrasonically treated 5 min before recording the photoluminescence emission spectra.

## Results and Discussion

**FIGURE 1:**
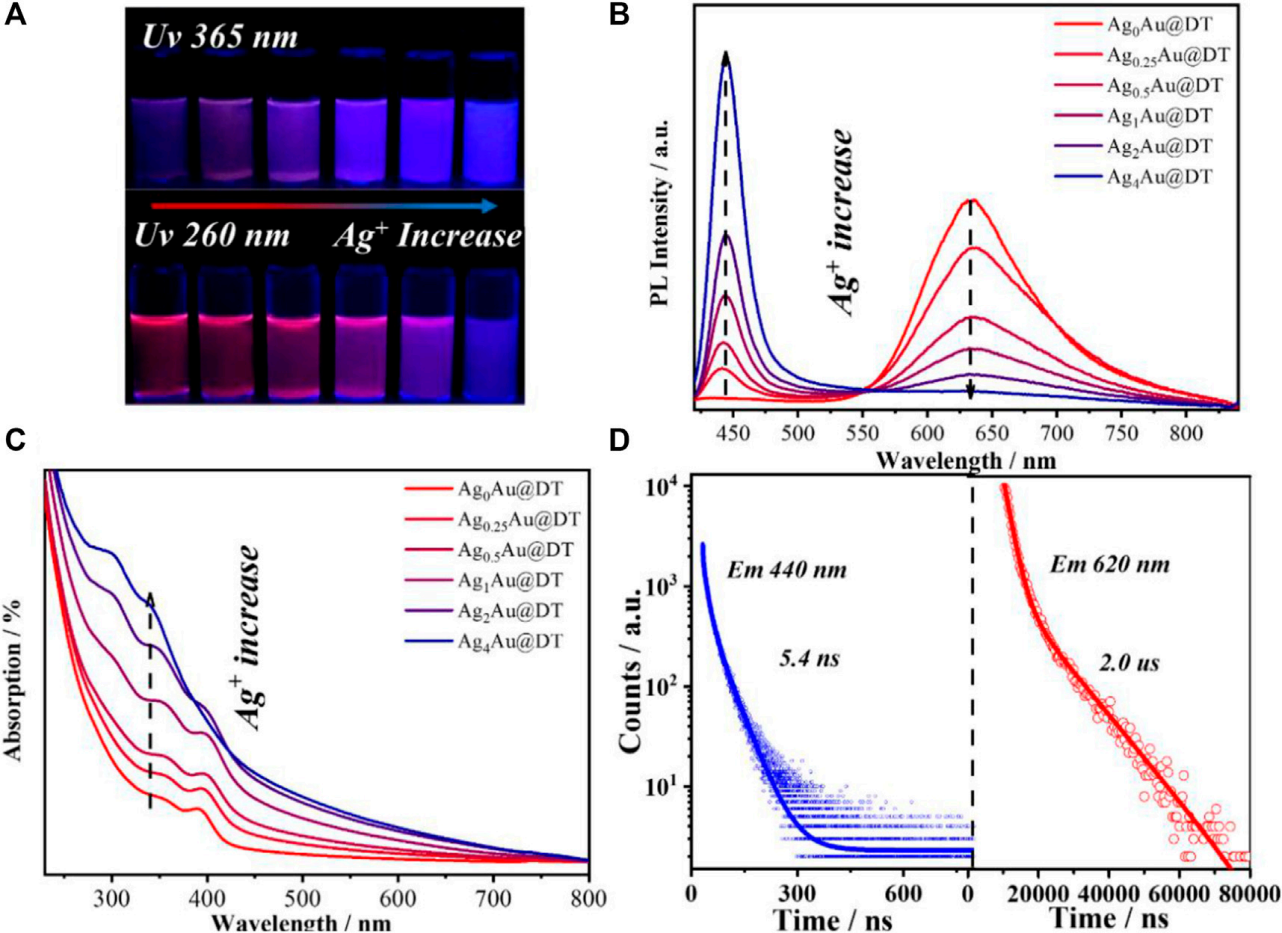
**(A)** Luminescent photographs of Ag_x_Au@DT NCs under UV 365 nm **(top)** and 260 nm **(bottom)** irradiations. Photoluminescence emission spectra **(B)** and absorption spectra **(C)** of Ag_x_Au@DT NCs. **(D)** Time-resolved luminescence decay profiles of Ag_1_Au@DT NCs measured at 440 nm **(left panel)** and 630 nm **(right panel)**, respectively.

The synthesis protocol of Au-Ag@DT NCs using DT as protected-cum-reduced ligand was modified from the previously reported method (Ye and An, 2019). Ag(I)-complexes were firstly formed by mixing DT alcoholic solution and AgNO_3_ under vigorous stirring for 10 min. HAuCl_4_ solution was then added into the mixed solution and the mixtures were further stirred for 12 h and incubated overnight at room temperature; the obtained samples were denoted as Ag_x_Au@DT NCs, in which the x represented the feeding ratio of Ag to Au. The incorporation of silver and gold to form Ag_x_Au@DT NCs was evidenced through the thermogravimetric analysis (TGA) and inductively coupled plasma (ICP) atomic emission spectroscopy (Supplementary Figure S1 and Supplementary Table S1), when increasing the feeding ratio of Ag-Au from 0.25, 0.5, 1, 2, and 4, the actual ratio of Ag to Au in the obtained NCs analyzed through ICP was determined as 0.11, 0.23, 0.47, 1.02, and 1.25, respectively, and the ligand weight loss (Supplementary Figure S1) was also gradually increased from 47.57 to 61.80%. Stoichiometric formula of obtained Ag_X_Au@DT NCs can be estimated as Ag_0_Au_1_DT_0.9_, Ag_0.1_Au_1_DT_1.2_, Ag_0.2_Au_1_DT_1.4_, Ag_0.5_Au_1_DT_1.5_, Ag_1_Au_1_DT_2.2,_ and Ag_1.3_Au_1_DT_2.7_ respectively (Supplementary Table S1), As shown in Figures 1A,B, when increasing the feeding ratio of Ag to Au, a new narrow blue emission band centered at 440 nm of Ag_x_Au@DT NCs was generated and then boosted, and the inherent red emission band centered at 630 nm of Au NCs was concomitantly declined, showing a relation of “as one falls, another rises” (Figure 1B). In a range of Ag/Au molar ratio from 0.25 to 2.0, because of the color mixing of dual-emissions, the as-synthesized Au–Ag bimetallic NCs exhibited the bright purple photoluminescence under UV irradiation of both 260 and 365 nm (Figure 1A). However, if further Ag^+^ doping with Ag/Au molar ratio of 4.0 was performed, the single emission at 630 nm of monometallic Au NCs almost completely disappeared, and only a sharp and strong emission band center at 440 nm was observed for Ag_4_Au@DT NCs (Figure 1B). Time-resolved PL spectroscopy showed that the radiation decay of blue and red emission channels had distinguished lifetimes of 5.4 nanoseconds (ns) and 2.0 microseconds (μs), respectively (Figure 1D). It is important to noe that, if according to conventional quantum size confinement mechanics of metal NCs, the inherent long-wavelength emission at 630 nm from Au core could not disappear since the Au NCs core remains intact with the increase of Ag^+^ loading (Supplementary Figure S2), suggesting the irrationality of Au NCs as emitters.

**FIGURE 2:**
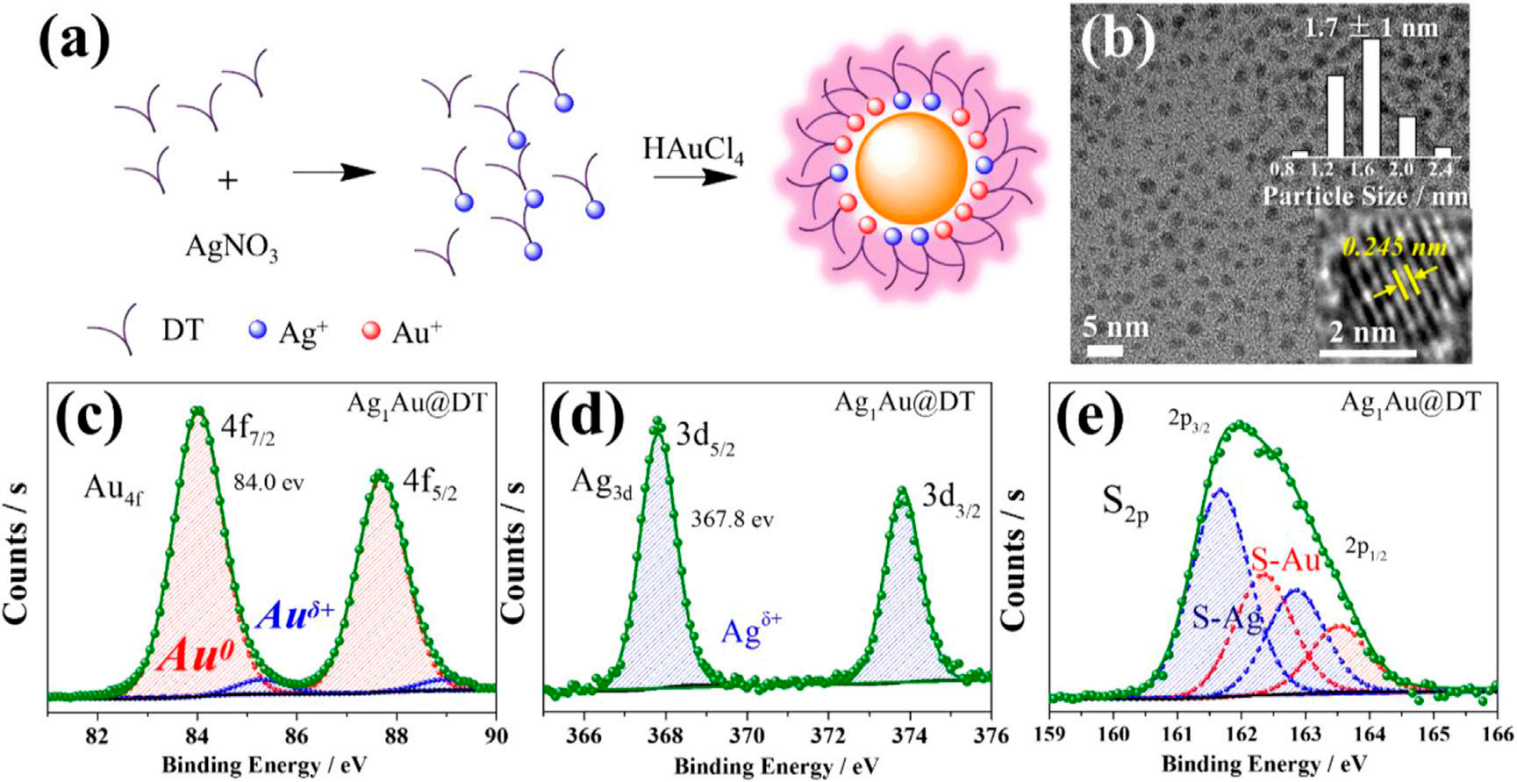
Scheme for the synthesis process of Ag_1_Au@DT NCs **(A)**, TEM **(B)**, inset, particle size distribution was determined by measuring the diameters of at least 100 metal NCs.) and XPS spectrum of Au 4f **(C)**, Ag 3 days **(D)** and S 2p (e) for Ag_1_Au@DT NCs.

The UV-visible absorption spectrum of Au_x_Ag@DT was collected in Figure 1C. Without the doping of silver, the Au NCs showed three remarkable absorption peaks located at 285 nm, 350 nm, and 390 nm, respectively, which has been previously assigned to Au 6sp intraband and interband transitions of Au NCs with 10–12 atoms (Negishi et al., 2005). The absence of localized surface plasmon resonance bands ranged at 400–520 nm, characteristic of plasmonic gold and silver nanocrystals or their alloy in the absorption spectrum, which implies that the particle size of Au–Ag bimetals is in the range of metal nanoclusters (Zhang et al., 2008), consistent with the results of TEM (Figure 2B and Supplementary Figure S2). Interestingly, although different chemical elements of silver were introduced into the system, with the increasing feeding ratio of Ag to Au, the peak positions of absorption bands at 285 nm, 350, and 390 nm were not shifted, while their intensity was gradually intensified, especially for the adsorption at 285 nm with significant improvement. Thus, we assigned the absorptions at 285 nm to be attributed to the surface ligand (including thiolate and hydrous hydroxyl groups, the latter comes from the adsorption of residual water in solution or air during the preparation of metal NCs) to metal (Au^+^ or Ag^+^) charge transfer (LMCT), consistent with an ascending trend of molar ratio of DT/Ag in Au-Ag bimetal NCs (Supplementary Figure S1 and Supplementary Table S2). The assignment of bands at 350 and 390 nm will be discussed later. Thus, we supposed that the Au-Ag bimetal NCs had a core–shell structure with Au NCs core coated by Au- and/or Ag-thiolate motifs (Figure 2A).

High-resolution transmission electron microscopy (HRTEM) and X-ray photoelectron spectra (XPS) characterizations supported this hypothesis. HRTEM images (Figure 2B) corroborated the size of as-formed Ag_1_Au@DT NCs of *ca.* 1.7 nm and no variation with the doping of silver (Supplementary Figure S2). The measured inter-planar distance from fringes in HRTEM images is 0.245 nm (Figure 2B insert), corresponding to the (111) plane of the cubic phase structure of Au (JCPDS ID 04–0,784). The XPS was used to analyze the valence charge state of metal and composition of Au-Ag bimetal NCs (Figures 2C–E). As shown in the Ag 3 days spectra (Figure 2D), the oxidation state of silver was all Ag(I) components with binding energy of 367.8 eV, suggesting the single Ag(I)-thiolate species in the Ag_1_Au@DT NCs (Dou et al., 2014b), and the Au 4f spectra (Figure 2C) were deconvoluted into Au(I) and Au (0) components with binding energies of 84.0 and 85.2 eV, respectively, and the content of Au(I) determined as such only accounts for a small fraction (∼5%) of all Au atoms in the Ag_1_Au@DT NCs. As reference to conventional Au-thiolate NCs (Zhou et al., 2010; Luo et al., 2012), this value is so small probably due to the substitution of Au(I)-thiolate staple motifs by incoming Ag(I)-thiolate and/or subsequent occurrence of reduction.

**FIGURE 3:**
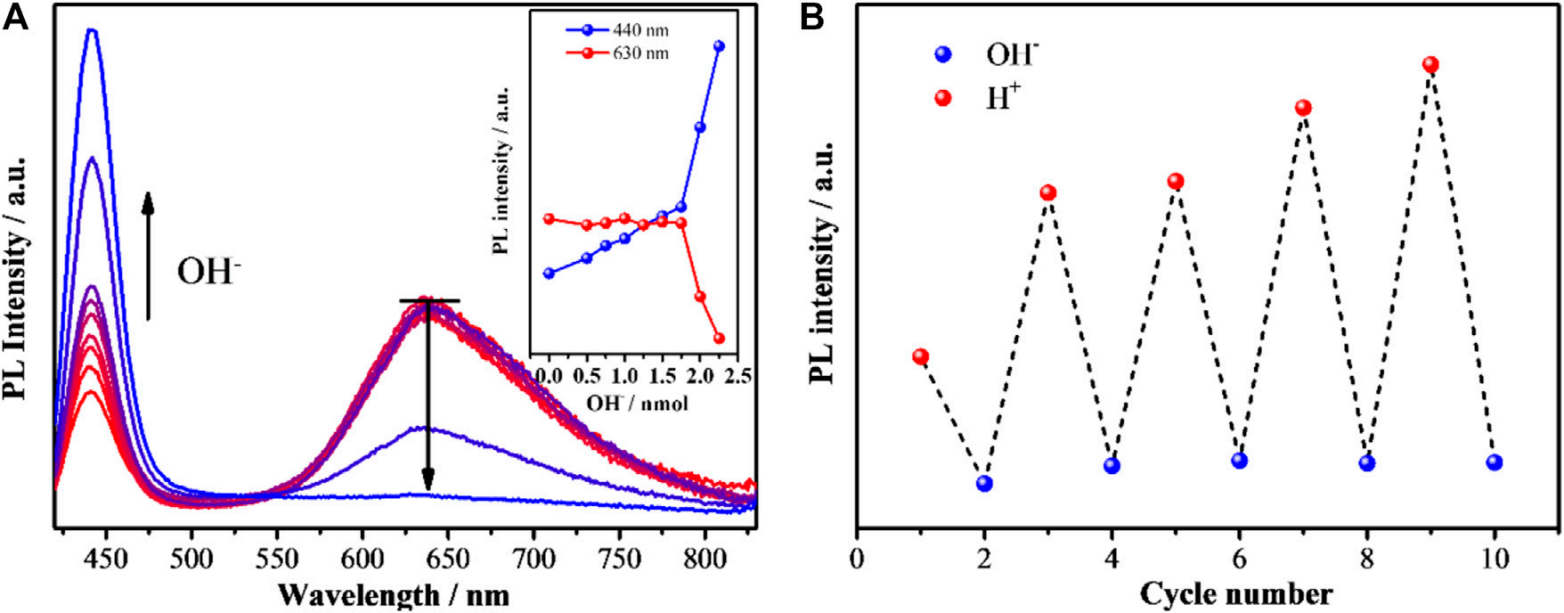
**(A)** Photoluminescence spectra of as-synthesized Ag_1_Au@DT NCs after adding increasing amounts of 50 mm NaOH aqueous solution. Fluorescence spectra were measured at λ_ex_ = 320 nm. The inset displays the relationship between photoluminescence (440 nm and 630 nm) intensity and the added mole amount of NaOH. **(B)** PL intensity of Ag_1_Au@DT NCs upon cyclic switching of the pH by adding 50 ul 50 mM NaOH and 50 ul 50 mm HCl aqueous solution.

More prominent results were demonstrated by the deconvolution of S 2p spectra (Figure 2E), two distinct components with binding energies of 161.7 and 162.3 eV were assigned to the Au(I)-thiolate and Ag(I)-thiolate according to soft-hard acid-base theory, respectively (Lu and Chen, 2012). The content of the latter one was increased with the increasing feed ratio of Ag to Au (Supplementary Figure S3 and Supplementary Table S2), which is consistent with the ICP and TGA results. The detailed fitting result is summarized in Supplementary Table S2. Therefore, a scheme was illustrated for the formation process of core-shell structured Ag_1_Au@DT NCs. Initially, Ag(I)-thiolate complexes were formed after the mix of DT and AgNO_3_; due to the lower redox potential of Ag^+^/Ag (0.8 eV) than AuCl_4_^−^/Au (1.0 eV) and the fast etching kinetics of Ag(I)-thiolate (Yuan et al., 2013), Ag(I)-thiolate was not reduced in this synthetic protocols, and the subsequently added HAuCl_4_ was reduced and protected by DT to form the metallic Au (0) core which stapled Ag(I)-thiolate and Au(I)-thiolate motifs together. Obviously, according to the well-accepted quantum size confinement effect of metal NCs, the short-wavelength emission at 440 nm cannot be answered by the surface nonmetal thiolate-Ag^+^ motif, and also we cannot understand why the emission at 630 nm was quenched with the increase of Ag^+^ and OH^−^ loading (Figures 1B, 3). Overall, our results of dual-emission of Au–Ag bimetal NCs mediated by Ag^+^ doping cannot support the quantum size confinement mechanics for the elucidation of origin of PL of metal NCs.

Not only the dosing of Ag^+^, but also the environmental pH of Ag_1_Au@DT NCs shows a prominent impact on the photoluminescence emission. Based on the theoretical calculation of H^+^ concentration production by the reduction of HAuCl_4_ precursor itself and exchange reaction of Au(I)/Ag(I) with thiol groups (-SH) during the synthesis of metal NCs, the amount of H^+^ in the stock solution can be precisely calculated to be 1.875 nmol (Eqs 1, 2, SI), so the solution of as made Au-Ag bimetallic NCs is acidic. Very interestingly, when varied amounts of NaOH solution (0, 0.5, 0.75, 1.0, 1.25, 1.5, 1.75, 2.0, and 2.25 nmol) were added, the dual-emissive Au-Ag NCs exhibited unique ratiometric pH-dependent emissions (Figure 3). As shown in Figure 3A, if the added amount of OH^−^ (0–1.75 nmol) is less than the calculated value (1.875 nmol), the red emission at 630 nm is almost unchanged, whereas the blue one (440 nm) was slightly but linearly increased with the increasing amount of OH^−^. Once more NaOH solution was added (>1.875 nmol), *i.e.,* the solution is basic, the dual emissions of Au-Ag NCs exhibited the opposite trend: the red emission dramatically reduced and almost quenched, while the blue emission precisely increased two times, suggesting that the original red-emissive Au emitters due to the strong coordination interaction of OH^−^ with Au (Figure 5B) induce the formation of the blue emitters (Figure 5C) because of Au/Ag molar ratio of 1:1 (Figure 3A and inset). In addition, we observed that the blue and red emission can be reversibly recovered and quenched by a cycling titration of acid and base solution (Figure 3B), indicating the meta-stability of luminous centers (Figures 5B,C), and concomitantly suggesting that the dual-emission of Au-Ag metal NCs is not dependent on the nature of metal NCs (*i.e.,* the PL of metal NCs is not dominated by quantum size confinement mechanics).

**FIGURE 4:**
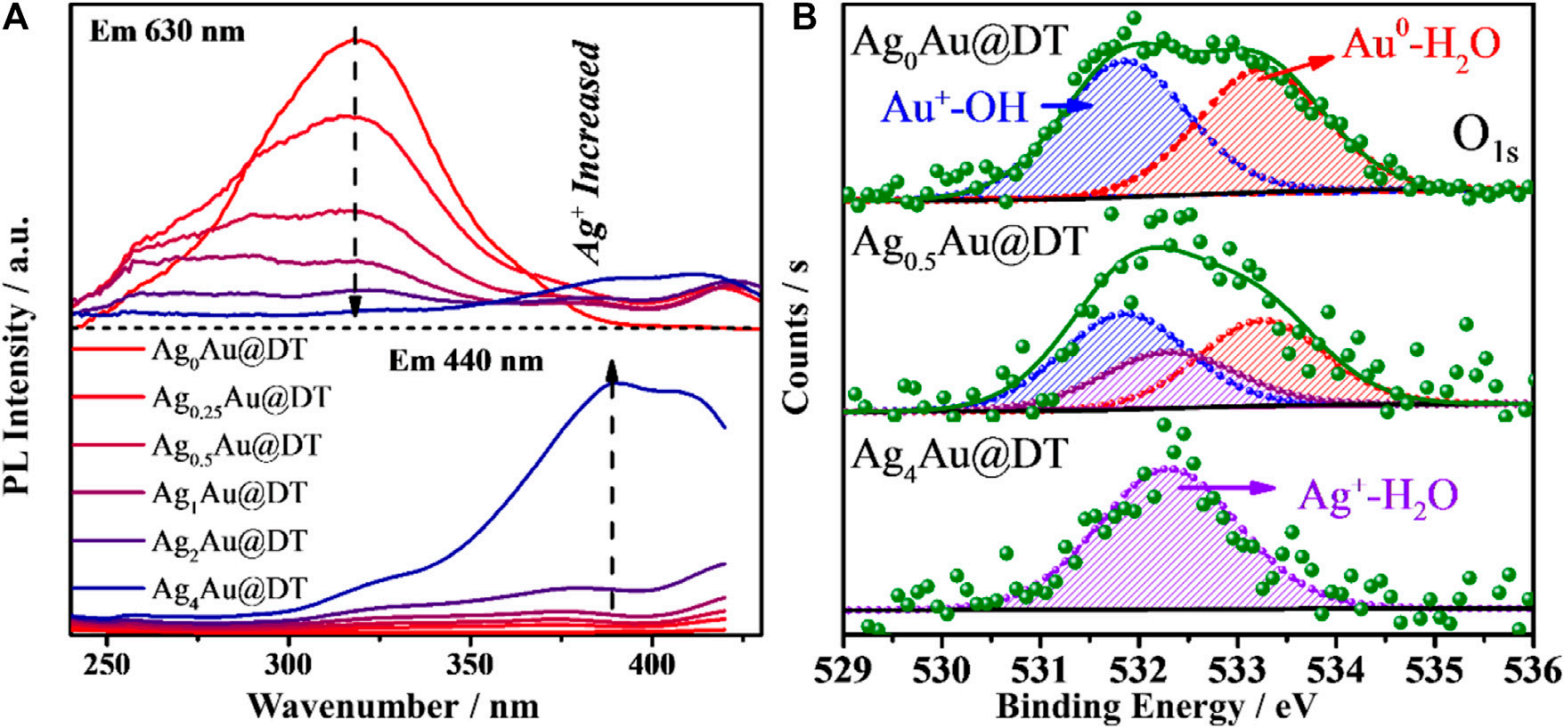
Excitation spectra **(A)** of Ag_x_Au@DT NCs and XPS spectrum **(B)** of O 1s for Ag_0_Au@DT and Ag_0.5_Au@DT and Ag_4_Au@DT NCs.

Nevertheless, the origin of dual-emission band for Ag_1_Au@DT NCs and its pH-dependent PL emission remains elusive. In most cases, the pH-dependent fluorescence properties were generally observed for ligand-protected metal NCs with AIE characteristics (Wu et al., 2019b; Chang et al., 2017; Su and Liu, 2017), in which the pH value was deemed to alter the charge state of the protected ligand and inhibited the aggregation of the Au(I)-thiolate staple due to the electrostatic repulsion. In our case, the pH variation cannot be a trigger of AIE for Ag_1_Au@DT NCs because of the nonionic nature of alkylthiol (DT) protective ligands on the metal core surface. In the previous discussion, we precluded the possibility of PL emission due to the quantum size confinement effect of metal NCs, since the Ag^+^ doping and pH variation did not change the structure and size of Au NCs core based on the characterizations of TEM and XPS (Supplementary Figures S2, S3). Excitation spectrum of Au-Ag bimetal NCs gives more information on the nature of excited states of dual emissions (Figure 4A). Unexpectedly, the long-wavelength red emission at 630 nm shows a broad excitation band centered at *ca.* 320 nm, and with an increase of Ag^+^ dosing, its excitation intensity is gradually decreased and finally completely disappears, consistent with the evolution of PL emission (Figure 1B); the short-wavelength blue emission at 440 nm exhibits a long-wavelength excitation centered at 390 nm. Similar excited state energy levels with just a 0.70 eV energy gap accounts for the strong energy transfer between two emission centers of Au-Ag bimetal NCs (Figure 1A), which also precludes the LMCT and/or LMMCT model of ligand to metal (or metal NCs) charge transfer for the origin of PL emission with much higher theoretical energy levels (Yang et al., 2019a), since pH did not significantly affect the coordination states of thiolate ligands with metals.

**FIGURE 5:**
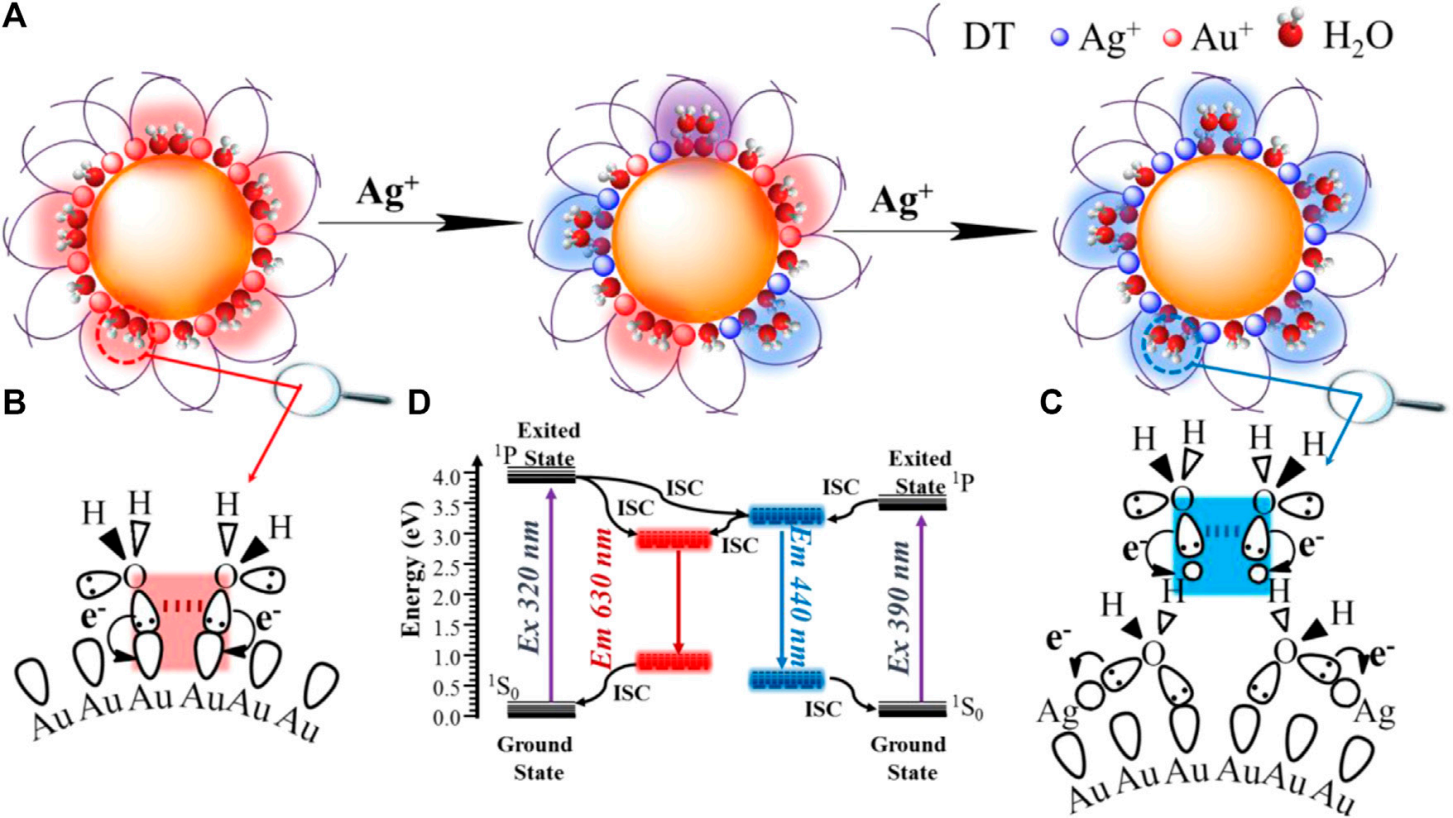
Schematic illustration of structural water molecules (SWs) confined at nanoscale interface of Au–Ag bimetal NCs packed by protective ligands emit bright dual-photoluminescence **(A)**, the red emissive SWs strongly binding on the single metal Au NCs **(B)**, the blue emissive SWs weakly binding on the fully core-shell structured Au-Ag bimetal Au NCs though water bridge which strongly coordinated with Ag^+^ **(C)**, and energy diagram of dual emissions of Au-Ag bimetal NCs **(D)**.

Very recently, after long-term and systematic investigation, using a combined experimental and computational approach, in particular with the help of steady and ultra-fast absorption and emission spectra, we unambiguously confirmed the existence of new interface states due to the spatial overlapping of *p* orbitals of oxygen atoms of structural water molecules (SWs) at the confined nanointerface of soft and hard nanocavity (Hu et al., 2020; Yang et al., 2020b; Yang et al., 2019a; Chen et al., 2014; Yang et al., 2017; Yang et al., 2019b; Yang et al., 2019c; Hu et al., 2021; Shan et al., 2021; Tao et al., 2021).It is called the *p* band intermediate state (PBIS) with *π* bonding features, and not only provides an ensemble of intermediate states for bright photoluminescence (PL) emission, but also acts as an alternative reaction channel for static electron transfer (Hu et al., 2021; Shan et al., 2021; Tao et al., 2021). Considering the ubiquitous properties of structural water molecules (SWs) at heterogeneous interface and the universality of our PBIS model, we performed the XPS measurement of O_1S_ to test the presence of species of SWs for three typical metal NCs of Ag_0_Au@DT and Ag_0.5_Au@DT and Ag_4_Au@DT NCs (Figure 4B). As expected, mono-metal Ag_0_Au@DT NCs show two distinct binding energies at 531.8 and 533.2 eV with molar ratios close to 1:1 (Figure 4B, top and Supplementary Table S3), which was assigned to hydroxide and water molecules chemically adsorbed on the Au(I) ion and Au atoms, respectively, indicating the presence of SWs {OH^−^∙H_2_O} at the metal core surface (Figure 5B). With the increase of Ag^+^ dosing in the synthesis, the content of SWs interacting with Au atoms was significantly decreased, while a new chemically adsorbed species containing O atoms of Ag_0.5_Au@DT was produced at 532.3 eV, which was assigned to water coordinated with Ag^+^ (Figure 4B, middle). If the Au core was completely covered by a thiolate-Ag^+^ motif shell, only Ag^+^-H_2_O complexes were observed at 532.3 eV (Yang et al., 2021) (Figure 4B, bottom and Figure 5C).

It is important to note that, in our previous report, after the vacuum evacuation, due to the removal of SWs with weak interactions, the blue emission at 440 nm of Ag NCs was quenched, and if trace amounts of water was re-added, the blue emission could be recovered (Yang et al., 2021). This probably accounts for why Ag_4_Au@DT NCs only captured one signal of chemically adsorbed water molecules with Ag^+^ at 532.3 eV owing to high vacuum treatment during the XPS measurement, indicating the blue emissive SWs are weakly bonded on the fully core-shell structured Au-Ag bimetal Au NCs though water bridge, which strongly coordinated with Ag^+^ (Figure 5C). Thus, PL properties strongly depend on the binding mode of SWs. Only the SWs with medium binding strength to metal emit the long-wavelength emission, while the SWs strongly coordinated with metals do not emit the color due to the limitation of spatial configuration of *p* oribtals of O atoms in the SWs, *i.e.,* {Ag^+^ H_2_O} dimers, but weakly bound SWs by hydrogen-Bonding on the {Ag^+^ H_2_O} dimers could emit the short-wavelength blue emission (Figure 5C). This also answers the origin of unique ratiometric pH-dependent dual-emissions (Figure 3). Under acid conditions, the hydroxide adsorbed on the Au–Ag bimetal NCs was neutralized, and only red-emission was observed. Under basic conditions, the first layer of SWs weakly interacted with Au(I) and Au (0) were replaced by hydroxide groups with stronger coordination ability to metals, which can be used as new immobilized sites for hosting SWs with weak interaction by H-bonding interaction (the blue emissive SWs with less stability), consequently the blue emission was intensified (Figure 3A). Obviously, the overlapping degree of two O atoms in the varied SWs, i.e., the stability of two type of SWs as emitters, determines the abnormal relation of long-wavelength emission with short-wavelength excitation (Figures 4A, 5) and their varied lifetimes of dual-emissions (Figure 1D), and this also answers the physical origins of two adsorption bands at 350 and 390 nm with corresponding excitation bands at 390 and 320 nm with π→π* transition characteristic due to the space interactions of *p* orbitals of two neighboring O atoms. The presence of SWs with the varied stability and multiple intermediate states at nanoscale interface probably answers the promoting role of alkali metal ion and hydroxyl group on the electro-catalytic reaction of water splitting (HER and OER), the selective reduction of CO_2_ and α, β-unsaturated aldehydes, and the water-gas shift reaction (WGSR) by providing the alternative channels for concerted proton and electron transfer (Hu et al., 2021; Shan et al., 2021; Tao et al., 2021). In addition, due to the strong overlapping of *p* orbitals between two oxygen atoms in the SWs at confined nanoscale interface, the polarity of O-H bonds in individual water molecules is significantly decreased, even close to zero, which accounts for the anomalously low dielectric constant of water confined under extreme confinement (Fumagalli et al., 2018; Coudert, 2020; Coudert et al., 2021; Geim, 2021).

The diagnostic experiments through dosing with S^2-^ anions (Supplementary Figure S4) demonstrated that the blue emission of Ag_1_Au@DT NCs was gradually quenched with the increased dosing amount S^2-^ due to its strong capacity to precipitate Ag^+^. Obviously, the removal of surface Ag^+^ induces the loss of SWs confined on the thiolate-Ag^+^ motifs, resulting in the quenching of blue emissions. Even though the red-emission was also reduced, its emission could be easily distinguished due to relatively strong affinity of SWs with Au(I) and Au (0) (Supplementary Figure S4 and inset). The weakening of red-emission is probably attributed to the covering of emitters due to the deposition of AgS precipitates. Indeed, the surface thiol ligand plays a role in creating the nano-microenvironment with hydrophobicity for hosting the SWs. This was verified by changing the DT concentration during synthesis to influence the dual-emissions (Supplementary Figure S5). At low concentrations of DT ligands (less than 2.0 equivalent to metal), the dual-emissions were hardly distinguished (Supplementary Figure S6, inset). Only above this critical value were the dual-emissions of Ag–Ag bimetal NCs boosted with more feeding of DT molecules, since the dense packing of DT on the metal core creates a more hydrophobic environment, which strengthens the stability of SWs (or stronger overlapping of *p* orbitals of O atoms by space interactions). Thus, we clearly addressed that the emitter of dual-emissive Au–Ag bimetal NCs are coming from the SWs with varied stability (Figures 5B,C), instead of metal NCs or AIE of surface protective ligands (Dou et al., 2014b; Wu et al., 2020).

If we consider SWs confined at the nanoscale interface as emitters, the reported abnormal PL properties of metal NCs could be easily elucidated, including solvent effect, pH-dependent behavior, surface ligand effect, multiple emitter centers, and large-Stoke’s shift, (De Cremer et al., 2009; Wu and Jin, 2010; Díez et al., 2012; Luo et al., 2012; AbdulHalim et al., 2014; Jia et al., 2014; Lin et al., 2014; Coutiño-Gonzalez et al., 2015; Wu et al., 2015; Fenwick et al., 2016; Yang et al., 2017; Aghakhani et al., 2018; Grandjean et al., 2018; Yang et al., 2019b; Hu et al., 2020). Herein, using our theory, taking an example of abnormal anti-galvanic replacement reaction approach for the synthesis of thiolate-protected Ag–Au NCs could be easily explained (Choi et al., 2010; Wu, 2012). Classical galvanic replacement reaction involves the spontaneous reduction of a noble-metal cationic by a less noble metal in solution driven by the difference in redox potentials (Ag + Au^+^→Ag^+^ + Au) (Udayabhaskararao et al., 2012; Gan et al., 2018), while the anti-galvanic replacement reaction means an inverse process (Ag^+^ + Au→Ag + Au^+^), which really subverted our traditional cognitive method. According to our model, in fact, this reaction did not happen through anti-galvanic replacement reaction. But, the intermediate states formed by SWs with the overlapping of *p* orbitals of O atoms provide the alternative channels for the reverse electron transfer between Au and Ag^+^, where the lost electron form Au (0) promotes the reduction of H^+^ (or adsorbed CO_2_ from air), while the oxidation of OH^−^ transfer the electron to Ag^+^, yielding the Ag (0) (Shan et al., 2021). The net reaction is water splitting with undistinguished production of H_2_ and O_2_ at nanoscale interface in the open system, which also answers the origin of bimetal NPs catalyzed water splitting. But, the experimental phenomenon of anti-galvanic replacement is completely right (Jeon et al., 2013; Jiao et al., 2019; Cammarata et al., 2021). Indeed, our proposed model of SWs as emitters with transient states benefiting electron transfer provides a reliable explanation of why the Ag NCs or NPs are very easy to be oxidized under air atmosphere. The discovery of these dynamic interface states also provides a completely new insight to understand the nature of heterogeneous catalysis and/or interface state (bonding), beyond the conventional metal-centered d band theory. (Hu et al., 2021; Shan et al., 2021; Tao et al., 2021; Medford et al., 2015; Liu et al., 2018)^]^

## Conclusion

In summary, bimetallic Ag-Au@DT NCs with well-resolved dual-emissions (440 nm and 630 nm) were successfully achieved through a facial one-pot approach. The newly boosted blue emission is the consequence of the doping of silver atom as Ag(I)-thiolate into the staple motif site of Au NCs, which is confirmed by the XPS and TEM characterization. Very interestingly, Ag_1_Au@DT NCs can sense a reversible dual-emission signal change in the environmental pH, which enabled it as a potential ratiometric nanoprobe for pH sensing. The combined characterizations of absorption, excitation, and emission spectrum confirm that the true emitters of dual-emission of Ag_1_Au@DT NCs are structural water molecules (SWs) confined on the surface of metal NCs core with varied stability. This accounts for why the PL properties of metal NCs are extremely susceptible to the surrounding microenvironments of SWs, such as the dosing of Ag^+^, pH, and packing mode of surface ligands. Since the adsorption of SWs at the nanoscale interface is a ubiquitous phenomenon in nature, the concept of “SWs as emitters” could be used as a base model to elucidate the origin of PL of other low-dimensioned quantum dots, such as carbon, semiconductor, and perovskites type materials. In addition, the concept of “surface electronic states” formed by space overlapping of *p* orbitals of O atoms in SWS could act as an alternative channel for concerted electron and proton transfer, which provide new insights for an in-depth understanding of heterogeneous catalysis and/or the nature of interface states (or interfacial bonding) (Hu et al., 2020; Hu et al., 2021; Shan et al., 2021; Tao et al., 2021).

## Data Availability

The original contributions presented in the study are included in the article/Supplementary Material, further inquiries can be directed to the corresponding authors.
