# Supplementary material for: Physical Origin of Dual-Emission of Au–Ag Bimetallic Nanoclusters
Source: Front Chem. 2021 Sep 27;9:756993. doi: 10.3389/fchem.2021.756993 (PMC8503609; doi:10.3389/fchem.2021.756993)
Supplement: Supplementary file 1 [file DataSheet1.docx]

Physical origin of dual-emission of Au–Ag bimetallic nanoclusters

Supplementary Material

# Supplementary Figures

**Supplementary Figure S1.** Thermalgravimetric analysis (TGA) of AgxAu@DT NCs with different ratio of Ag to Au.


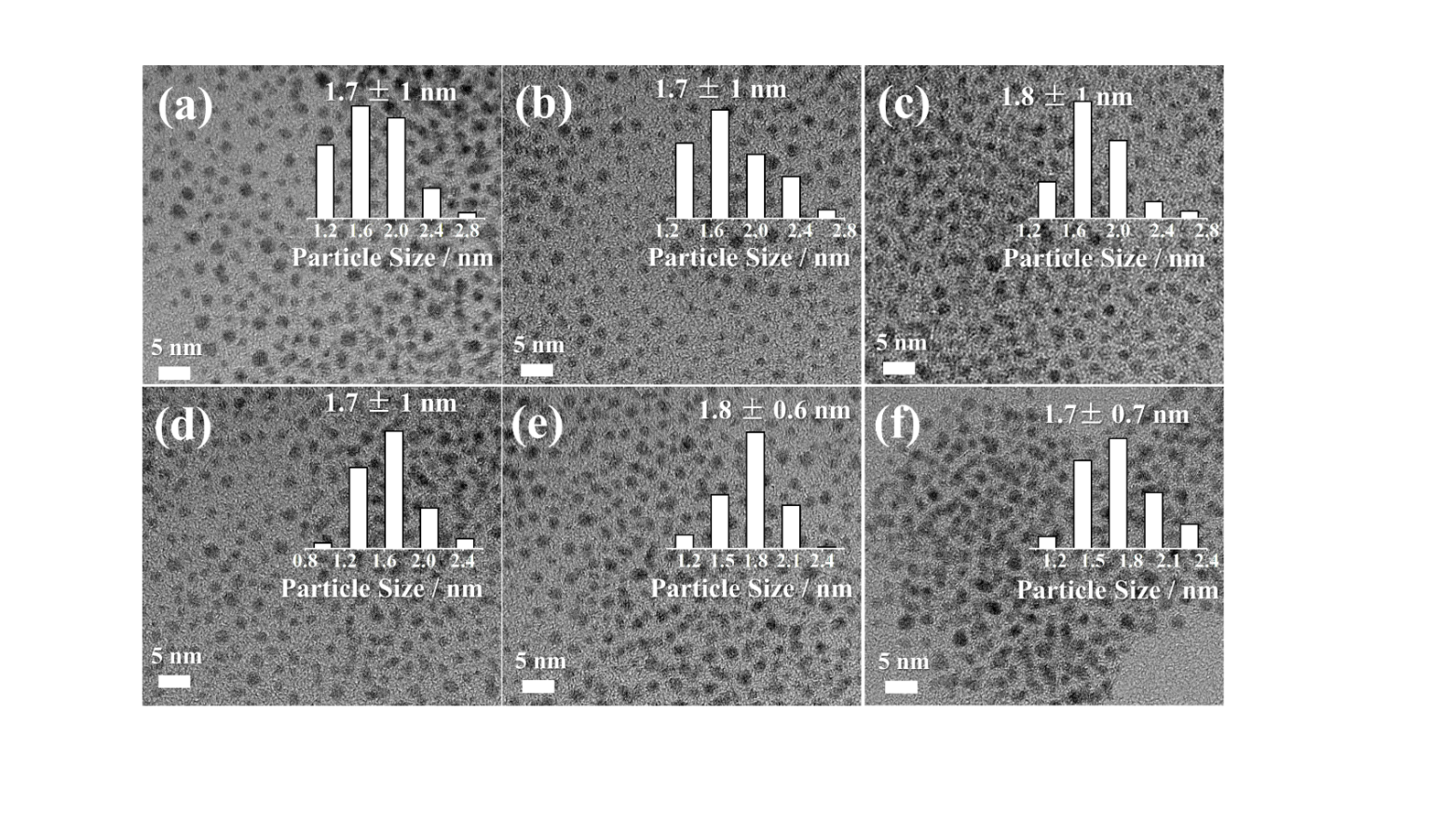


**Supplementary Figure S2.** HRTEM micrograph of Ag_0_Au@DT (a), Ag_0.25_Au@DT @DT (b), Ag_0.5_Au@DT (c), Ag_1_Au@DT (d), Ag_2_Au@DT NCs (e) and Ag_4_Au@DT NCs (f), respectively. The scale bar is 10 nm. The inset shows the size distribution.


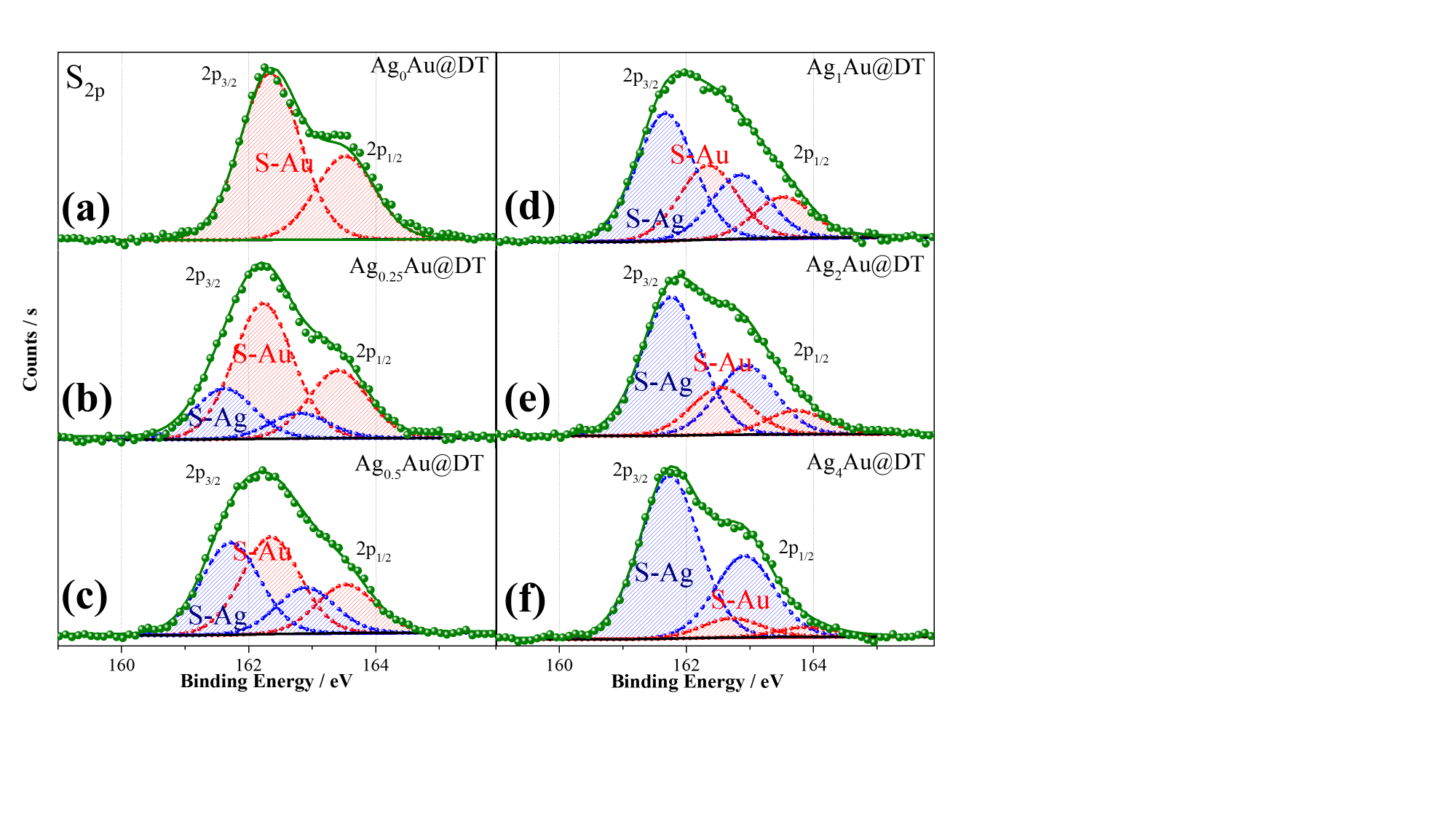


**Supplementary Figure S3.** XPS spectrum of S 2p for Ag_0_Au@DT (a), Ag_0.25_Au@DT @DT (b), Ag_0.5_Au@DT (c), Ag_1_Au@DT (d), Ag_2_Au@DT NCs (e) and Ag_4_Au@DT NCs (f), respectively.


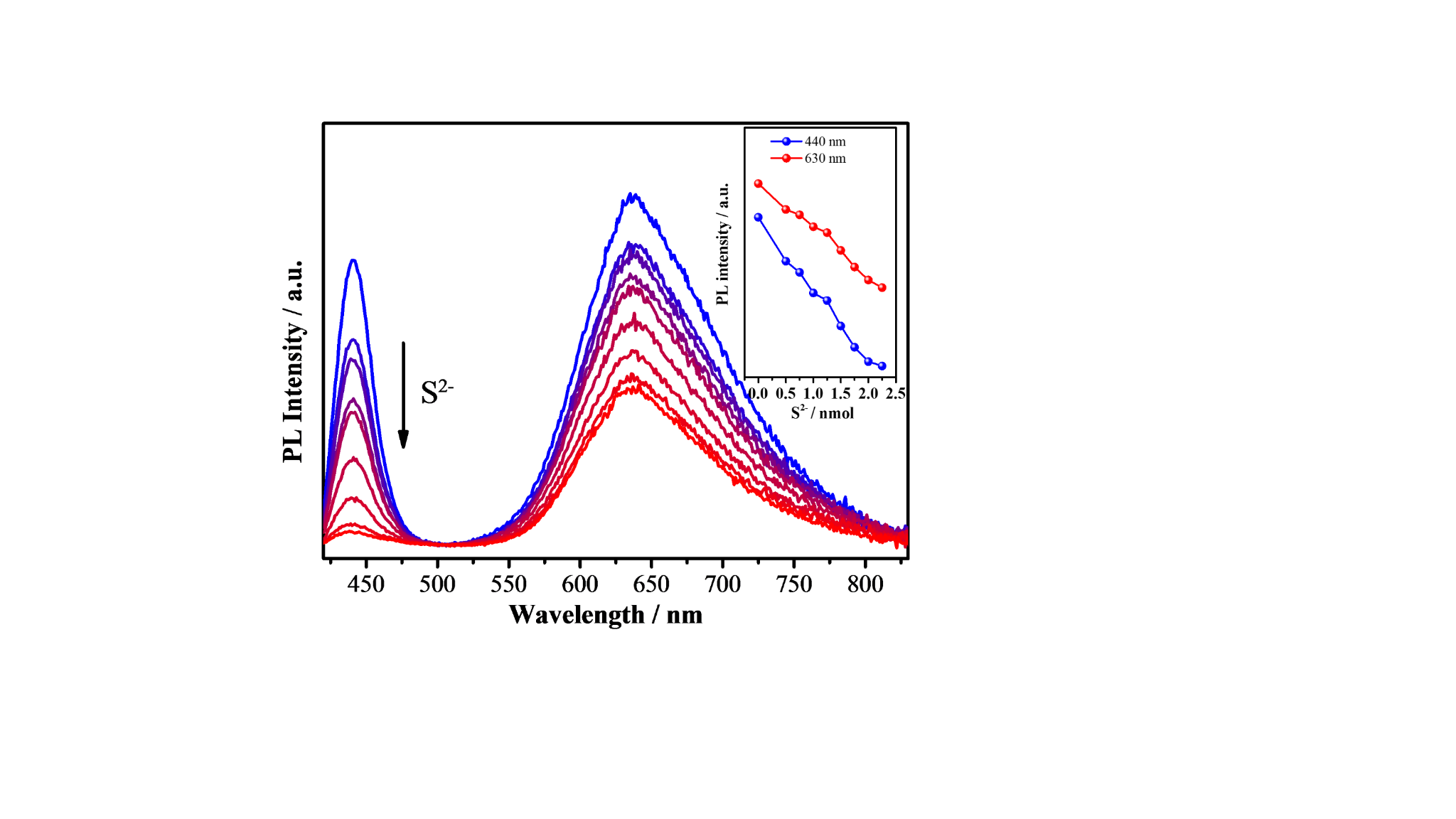


**Supplementary Figure S4.** Photoluminescence spectra of as-synthesized Ag_1_Au@DT NCs after adding increasing amount (0, 0.5, 0.75, 1.0, 1.25, 1.5, 1.75, 2.0 and 2.25 nmol) of 50 mM K_2_S aqueous solution. The inset displays the relationship between photoluminescence (440 nm and 630 nm) intensity and the added mole amount of K_2_S.


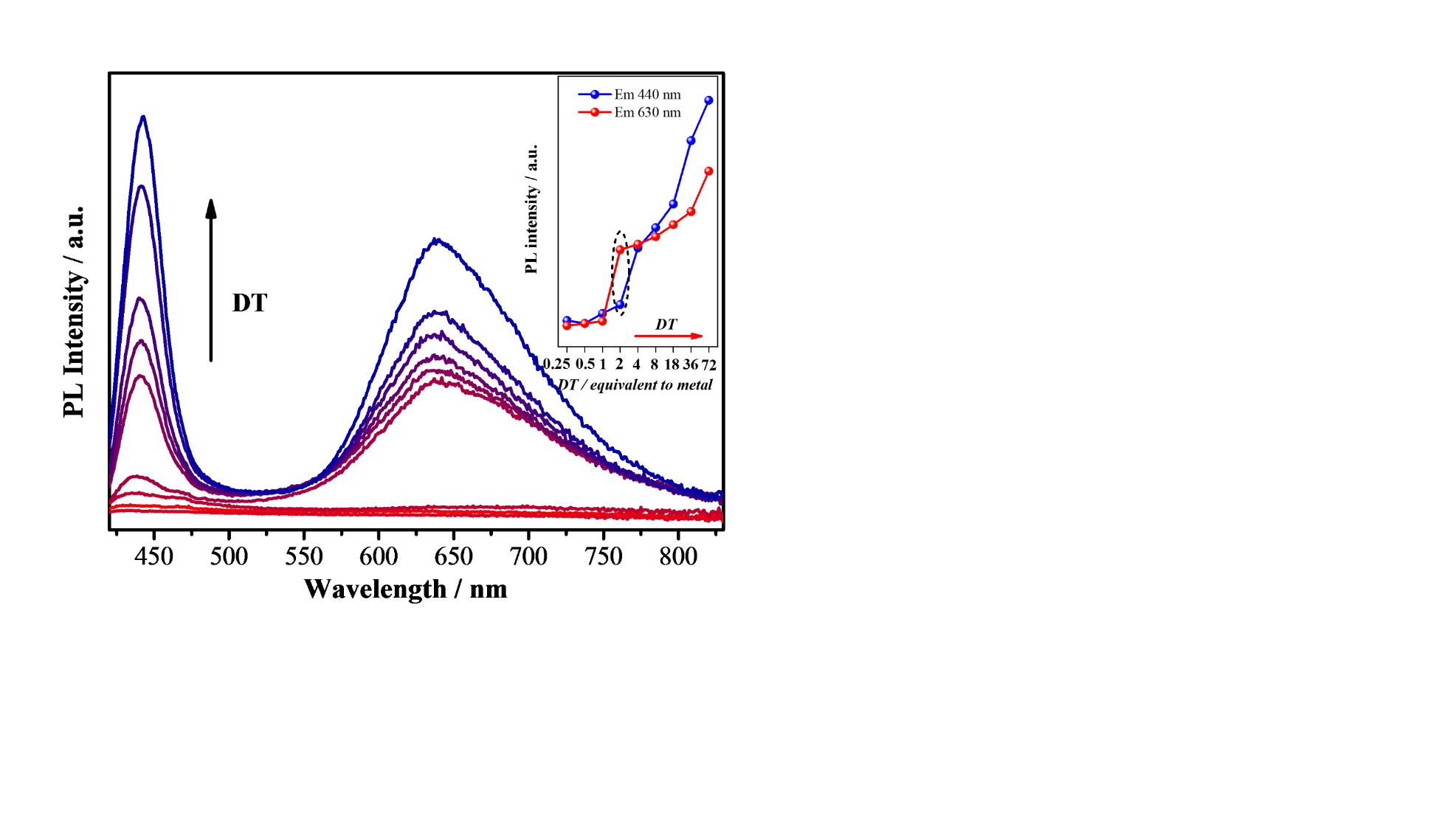


**Supplementary Figure S5.** PL emission spectra of Ag_1_Au@DT NCs synthesized at increasing DT concentration (0.25, 0.5, 1, 2, 4, 8, 18, 36 and 72 equivalent to metal, respectively). The inset displays the relationship between photoluminescence (440 nm and 630 nm) intensity and the amount of DT equivalent to metal.

# Supplementary Tables

## Supplementary Table S1. The ratio of Ag to Au of Ag_x_Au@DT NCs determined by ICP-OES analysis.

| ***Sample*** | | ***The molar ratio of Ag-Au*** | | | ***Stoichiometric Formula^a^*** |
| --- | --- | --- | --- | --- | --- |
|  |  | ***Feed*** | | ***ICP*** |  |
| ***Ag_0_Au@DT NCs*** | ***0*** | | ***-*** | | ***Ag_0_Au_1_ DT_0.9_*** |
| ***Ag_0.25_Au@DT NCs*** | ***0.25*** | | ***0.11*** | | ***Ag_0.1_Au_1_DT_1.2_*** |
| ***Ag_0.5_Au@DT NCs*** | ***0.5*** | | ***0.23*** | | ***Ag_0.2_Au_1_DT_1.4_*** |
| ***Ag_1_Au@DT NCs*** | ***1*** | | ***0.47*** | | ***Ag_0.5_Au_1_DT_1.5_*** |
| ***Ag_2_Au@DT NCs*** | ***2*** | | ***1.02*** | | ***Ag_1_Au_1_DT_2.2_*** |
| ***Ag_4_Au@DT NCs*** | ***4*** | | ***1.25*** | | ***Ag_1.3_Au_1_DT_2.7_*** |

^a^Stoichiometric formula was estimated by the calculation of DT to metal weight ratio (TG analysis) and Ag to Au molar ratio (ICP analysis) of Ag_x_Au@DT NCs.

**Supplementary Table S2.** XPS peak parameters for the S state (**Supplementary Figure S4**) of Ag_x_Au@DT NCs.

| ***Sample*** | ***Atom*** | ***Item*** | ***Position (eV)*** | ***Content (%)*** |
| --- | --- | --- | --- | --- |
| ***Ag_0_Au@DT NCs*** | ***S 2p*** | ***Ag(I)-SR*** | ***161.6*** | - |
|  |  | ***Au(I)-SR*** | ***162.3*** | ***100*** |
| ***Ag_0.25_Au@DT NCs*** |  | ***Ag(I)-SR*** | ***161.6*** | ***27.3*** |
|  |  | ***Au(I)-SR*** | ***162.2*** | ***72.7*** |
| ***Ag_0.5_Au@DT NCs*** |  | ***Ag(I)-SR*** | ***161.7*** | ***48.7*** |
|  |  | ***Au(I)-SR*** | ***162.3*** | ***51.3*** |
| ***Ag_1_Au@DT NCs*** |  | ***Ag(I)-SR*** | ***161.7*** | ***63.2*** |
|  |  | ***Au(I)-SR*** | ***162.3*** | ***36.8*** |
| ***Ag_2_Au@DT NCs*** |  | ***Ag(I)-SR*** | ***161.8*** | ***74.4*** |
|  |  | ***Au(I)-SR*** | ***162.5*** | ***25.6*** |
| ***Ag_4_Au@DT NCs*** |  | ***Ag(I)-SR*** | ***161.7*** | ***88.5*** |
|  |  | ***Au(I)-SR*** | ***162.7*** | ***10.5*** |

**Supplementary Table S3.** XPS peak parameters for the O (**Figure 4b**) states of Ag***_x_***Au@DT NCs

| ***Sample*** | ***Atom*** | ***Item*** | ***Position (eV)*** | ***Content (%)*** |
| --- | --- | --- | --- | --- |
| ***Ag_0_Au@DT NCs*** | ***O 1s*** | ***Au^+^-OH*** | ***531.8*** | ***52.4*** |
|  |  | ***Ag^+^-H_2_O*** | ***-*** | - |
|  |  | ***Au^0^-H_2_O*** | ***533.2*** | ***47.6*** |
| ***Ag_0.25_Au@DT NCs*** |  | ***Au^+^-OH*** | ***531.8*** | ***39.7*** |
|  |  | ***Ag^+^-H_2_O*** | ***532.3*** | ***25.0*** |
|  |  | ***Au^0^-H_2_O*** | ***533.2*** | ***35.3*** |
| ***Ag_0.4_Au@DT NCs*** |  | ***Au^+^-OH*** | ***-*** | ***-*** |
|  |  | ***Ag^+^-H_2_O*** | ***532.3*** | ***100*** |
|  |  | ***Au^0^-H_2_O*** | ***-*** | ***-*** |
